# Letter to the Editor: “The effect of chiropractic care on infantile colic: results from a single-blind randomised controlled trial” and “Identifying potential treatment effect modifiers of the effectiveness of chiropractic care to infants with colic through prespecified secondary analyses of a randomised controlled trial”

**DOI:** 10.1186/s12998-021-00386-1

**Published:** 2021-08-04

**Authors:** Braden Keil, Christian Fludder

**Affiliations:** Private Practice, Victoria Melbourne, Australia

We would like to acknowledge Holm et al.’s publication of their articles. However, multiple major limitations result in the outcomes being of little use in improving knowledge of infantile colic and manual therapy.

Firstly, manual therapy was researched not “chiropractic care”; the intervention is used by multiple professions [1].

The intervention used is poorly quantified; forces, rate of force and treatment duration were not recorded or described. Force measuring equipment is cheap and readily available; research should record intervention forces.

The two interventions were inadequately described preventing replication. How many received both or either? Was there an outcome difference? The conclusion that a significant effect was not observed is questionable.

Increased neurophysiological responses occur with sub-100ms thrust duration compared to the non-thrust intervention [2]. Our clinical experience and previous research support the non-significant effect found. Manual therapy taught in recognized post-graduate programs and used by Australian paediatric chiropractors differs significantly meaning the study outcome has minimal relevance to current practice and training.

Data relating to joints receiving the intervention was not provided. Another disappointing omission. Previous research demonstrated very high association of shoulder and atlantooccipital joint dysfunction in irritable infants [3].

The control group received an intervention likely to provide proprioceptive afferentation possibly associated with reduced pain, as well changes to Vagal tone [4]. The conclusion should be that the intervention was not significantly better than the compared control intervention. Whether either intervention is significantly better than no intervention remains unanswered.

Holm et al. used “chiropractors with a special interest and experience in paediatrics”. Detail regarding levels of training in paediatrics is needed. Australian post-graduate training in chiropractic paediatrics is accredited by the Australian College of Chiropractic Paediatrics (ACCP) with a mandated minimum 2 year, 1100-hour requirement. Did the chiropractors involved meet this minimum level of training? The conclusion applies only to chiropractors without recognized post-graduate paediatric training.

It is strange that items relating to developmental dysplasia of the hips (DDH) were included. DDH is not a painful syndrome and is not able to be treated by manual therapy. Clinically important markers such as head asymmetry, position and control with pull-to-sitting test, degree and symmetry of Moro reflex, hand fisting, symmetry of arm movement, and degree of vomiting were important omissions.

It is disappointing that we are still running clinical trials that are incomplete or lacking in detail. This study would have benefitted from input from chiropractors with post-graduate training in chiropractic paediatrics.

## Data Availability

Not applicable.
